# Isolation and molecular characterization of foot-and-mouth disease viruses from small ruminants in Nigeria

**DOI:** 10.1186/s13567-026-01809-8

**Published:** 2026-06-24

**Authors:** David O. Ehizibolo, Olumuyiwa Oyekan, Moses O. Oguche, Abdullahi Ardo, Anna B. Ludi, Britta A. Wood, Simon Gubbins, Hayley M. Hicks, Jemma Wadsworth, Etienne Chevanne, Fabrizio Rosso, Nick J. Knowles, Valerie Mioulet, Donald P. King, Georgina Limon

**Affiliations:** 1 National Veterinary Research Institute, Vom, Plateau State Nigeria; 2 The Pirbright Institute, Ash Road, Pirbright, Woking, UK; 3 European Commission for the Control of Foot-and-Mouth Disease, Food and Agriculture Organization of the United Nations, Rome, Italy

**Keywords:** Isolation, identification, FMD, FMDV, small ruminants, Nigeria

## Abstract

Foot-and-mouth disease (FMD) poses significant economic challenges to the livestock industry in Nigeria due to its widespread prevalence and detrimental impacts on animal health and international trade. Documented FMD outbreaks in the endemic areas of West and Central Africa consistently highlight the role of cattle as the indicator species for FMD. However, the role of small ruminants (SR) is poorly understood. Aiming to improve our understanding of the role of SR in the epidemiology of the disease, this study investigated FMD outbreaks on farms where cattle intermixed with sheep and goats. Blood and oral swabs (*n* = 177) were collected from 138 sheep and 39 goats in nine local government areas (LGAs) of Plateau State, Nigeria. Additionally, epithelial tissue samples were obtained from suspected cases of FMD in six sheep and three goats in four of the LGAs. The majority (71.2%, 95% CI 64–77) of SR sampled seroconverted to FMDV measured by a 3ABC non-structural protein (NSP) enzyme-linked immunosorbent assay (ELISA) where 77.5% (95% CI 70.7–84.5) of sheep and 48.7% (95% CI 33–64.4) goats tested positive for FMDV-NSP antibodies. FMDV genomic RNA was detected in 36.6% (95% CI 25–49) of oral swabs samples collected from SR and two FMDV serotypes: O and A were detected using antigen-ELISA. Phylogenetic analyses of the VP1 coding sequences indicate that these viruses were closely related to those identified from cattle in the same farms and to those previously published from the same region. The findings from this study demonstrate that SR should be considered when developing FMD risk-based surveillance and control strategies in Nigeria.

## Introduction

Foot-and-mouth disease virus (FMDV) is the causative agent of FMD, a highly infectious disease that impacts both domestic and wild cloven-hoofed animals. FMDV is classified within the RNA virus family *Picornaviridae* and the genus *Aphthovirus*. Notably, the virus exhibits considerable genetic and antigenic diversity [1, 2]. Globally, small ruminants (SR: sheep and goats) represent the largest population of domestic livestock that is vulnerable to FMD. However, the disease frequently goes unnoticed in sheep and goats due to its asymptomatic nature. Common clinical signs of FMD in SR such as lameness, fever, depression and neonatal mortality, lack disease specificity. Meanwhile, vesicular lesions, in and around the mouth, on the feet or other areas are less frequent in occurrence in SR [3]. Like other ruminant species, FMDV infection in sheep can result in a persistent subclinical infection, documented to persist for up to 9 months [4, 5], a duration comparatively shorter than that observed in cattle and buffalo [6, 7]. Therefore, SR can act as asymptomatic hosts of FMD and their contribution to the epidemiology of FMD is often underestimated and poorly understood due to the absence of noticeable symptoms [3, 8].

The importance of sheep in FMDV incursions into formerly FMD-free nations was underscored by the substantial spread resulting from the movement of infected sheep during the initial phases of the 2001 FMD epidemic in the UK [9]. However, the involvement of sheep in FMD epidemiology in areas where the disease is endemic remains largely unexplored, as sheep are frequently excluded from FMD surveillance or vaccination initiatives.

In Nigeria, SR production constitutes 84.5% of total grazing domestic livestock comprising 48.6 million sheep and 76.3 million goats [10], where the majority of these animals are concentrated in the northern region of the country. This sector is a primary source of meat and significantly contributes to the country’s food security. Indigenous breeds of sheep and goats are predominantly raised alongside cattle, especially sheep in the traditional extensive system practiced by pastoralists and agro-pastoralists in northern regions of Nigeria.

FMD is entrenched as an endemic disease in Nigeria constituting an ongoing challenge to the nation’s livestock sector. Diverse topotypes or genotypes of four FMDV serotypes (O, A, SAT 1 and SAT 2) have been documented, primarily affecting cattle [11–16]. Over the years, FMD outbreaks documented across West and Central Africa indicate that cattle are the dominant FMD-susceptible species in the region. Small ruminants are susceptible to FMDV infection, and since they are often a mobile population, their frequent movements could contribute to the dissemination of the disease within the country. FMD genomic RNA has been identified in sera of SRs, and serological evidence of specific FMDV-non-structural protein (NSP) antibodies has been reported in SRs in Nigeria [11, 17–21]. The objective of this study was to isolate and identify FMDV in SR, as part of outbreak investigations in Northern Nigeria, to help understand the role of these species in the epidemiology of FMD in West and Central Africa.

## Materials and methods

### Sample collection

**Figure 1:**
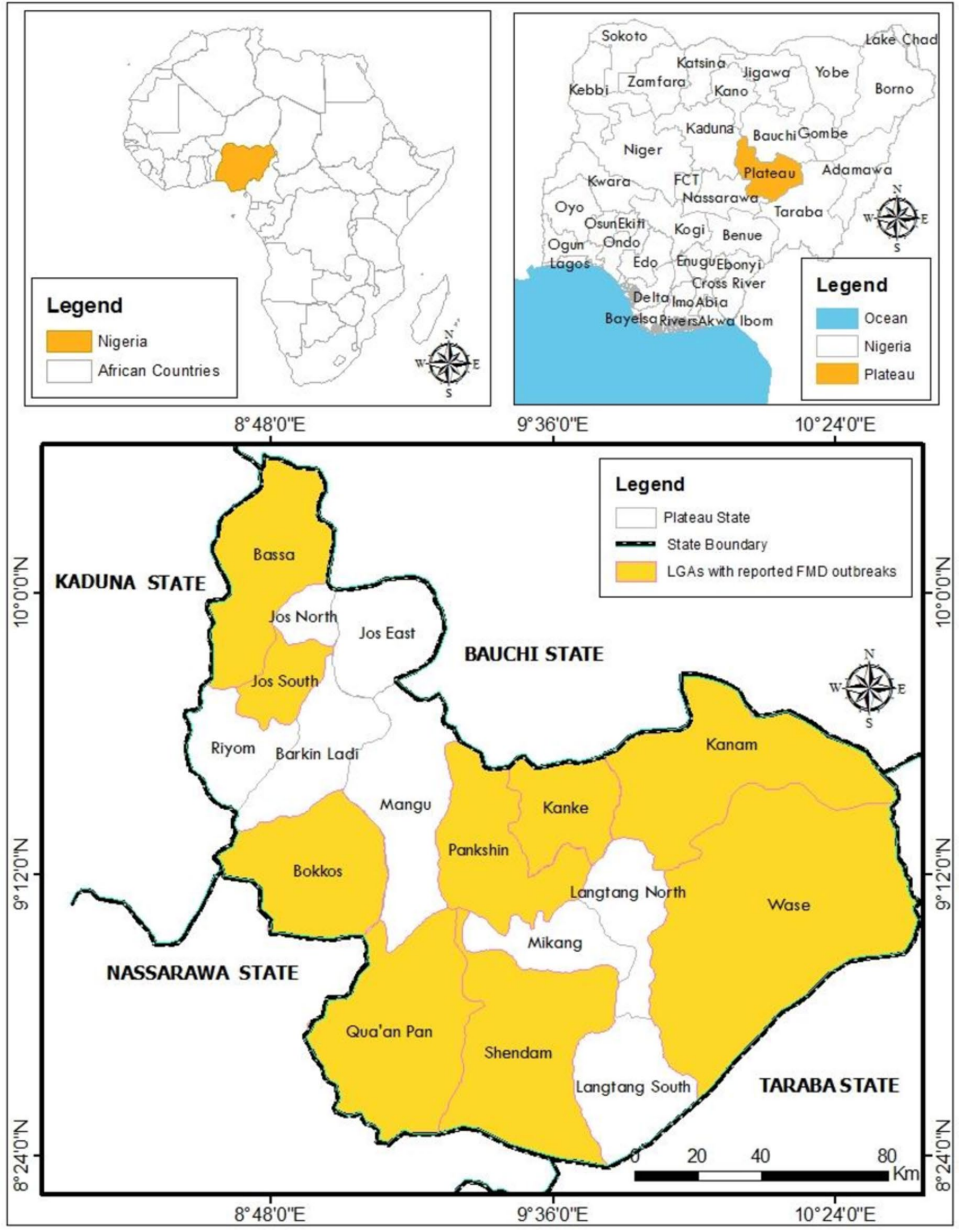
**Map showing the location of LGAs with reported FMD outbreaks.**

During the period from September 2020 to December 2021, Community Animal Health Workers (CAHWs) across 17 out of the 21 local government areas (LGAs) in Plateau state, Nigeria were engaged to collect information, and were tasked with monitoring and reporting outbreaks of FMD (Figure 1). Cases investigated in cattle herds intermixed with small ruminants across 9 LGAs were confirmed based on the presence of clinical signs in cattle. Paired samples comprising blood and nasal swabs (*n* = 177) were collected from 138 sheep and 39 goats. Furthermore, epithelial tissue samples were obtained from suspected cases of FMD in sheep (*n* = 6) and goats (*n* = 3) originating from Kanke, Bassa and Pankshin LGAs (for sheep) and Qua’anpan LGA (for goats). In each reported FMD case within cattle herds mixed with SRs, a thorough examination was conducted of the SRs to identify any clinical signs of the disease. Five millilitres of blood were drawn from the jugular vein of each chosen debilitated animal, utilizing pre-labeled vacutainer tubes, while a sterile swab stick was employed to swab the oropharyngeal/oral cavity, which was promptly placed into viral transport medium (VTM). Separated serum and oral swab samples were stored at −20 °C at National Veterinary Research Institute (NVRI) until tested. Meanwhile, the epithelial tissue samples were shipped to The Pirbright Institute, UK for analysis.

### Laboratory analysis

#### Serological analysis

The prioCHECK FMDV NS Antibody Test ELISA kit (Thermo Lelystad, The Netherlands) was used according to the manufacturer’s instruction for detection of antibodies to the highly conserved non-structural 3ABC protein of FMDV in all sera collected [22]. Sera that generated a percent inhibition greater than or equal to 50% were considered positive.

#### Virological analysis

##### Real-time reverse transcriptase-polymerase chain reaction (rRT-PCR)

The rRT-PCR was performed on sixty-six of the oral swab samples collected from sheep within infected cattle herds during outbreaks. In addition, only five oral swab samples from goats were selected for rRT-PCR analysis based on the suitability of the samples for molecular testing. The RNA from the samples was extracted using Qiagen RNA extraction kit (Qiagen, Hilden, Germany) according to manufacturer’s instructions. Eluted RNA was analyzed in a One-Step rRT-PCR (Cat no: 204343) targeting the FMDV RNA-dependent polymerase (FMDV 3D gene) as described by Callahan et al. [23]. Cycle threshold (Ct) values < 40 was considered positive.

##### Virus isolation and antigen ELISA

Epithelium samples were processed at the World Reference Laboratory for Foot-and-Mouth Disease (WRLFMD) (Pirbright, UK) following the method described in the WOAH manual [24]. Briefly, clarified sample homogenate was inoculated onto WRL-LFBK cells washed with PBS and cultured in Nunc^™^ cell culture tubes [25]. A 30-min adsorption step was carried out at 37 °C, 2 mL media (MEM; Life Technologies supplemented with 6 mL/L in-house antibiotics) were then added and the tubes were incubated at 37 °C with slow rotation for up to 4 days, with daily microscopic examination for the presence of cytopathic effect (CPE). Where CPE was observed, supernatants were clarified by centrifugation at 3000 *g* for 10 min at 4 °C. An aliquot was then tested by antigen-detection ELISA [26] according to procedures described in the FMD Chapter of the WOAH Terrestrial Manual [24].

#### Sequences and phylogenetic analysis

VP1 sequences were determined at the WRLFMD using the method described by Knowles et al. [27]. Specifically, two independent RT-PCR assays were conducted for each serotype. For serotype O, the primer pairs used were O-1C244F (5'-GCAGCAAAACACATGTCAAACACCTT) or O-1C272F (5'-TBGCRGGNCTYGCCCAGTACTAC) in combination with EUR-2B52R (5'-GACATGTCCTCCTGCATCTGGTTGAT). For serotype A, the primer pairs were A-1C562F (5'-TACCAAATTACACACGGGAA) or A-1C612F (5'-TAGCGCCGGCAAAGACTTTGA) along with EUR-2B52R. Sanger sequencing was conducted using an ABI 3730xl DNA Analyzer (ABI Biosystems, Waltham, Massachusetts). The assembled VP1 sequences of FMDVs from sheep and goats in this study, along with strains of serotypes O and A from Nigeria obtained during the outbreak period from cattle in the same farms, were compared with sequences retrieved from the NCBI GenBank database. Raw sequence data were assembled using Lasergene SeqMan Pro version 17.3.0 (DNAStar Inc.) prior to downstream analysis. Multiple sequence alignments were performed using Clustal W [28] within the MEGA 11 software [29] to classify the virus by serotype, topotype, and lineage. The phylogenetic tree was built using MEGA 11, employing the Maximum Likelihood method with 1000 Bootstrap confidence values. The best nucleotide substitution model was determined to be the Tamura 3-parameter model using a discrete Gamma distribution to model evolutionary rate differences among sites. Additionally, the nucleotide sequence identities of the VP1 sequences were compared. The VP1 sequences of five isolates from SR reported here for the first time in Nigeria were submitted to GenBank along with 69 sequences from viruses isolated from cattle (accession numbers: PQ381806-PQ381879).

### Statistical analysis

Seroconversion to FMDV antibodies was summarized as proportions across animal species. Differences in seropositivity between species were evaluated using the chi-square test of independence. Proportions of seropositive animals were estimated with corresponding 95% confidence intervals using the normal approximation method [30]. A p-value of less than 0.05 was considered statistically significant.

## Results

Overall, 126 out of 177 sera tested positive (71.2%, 95% CI 64–77). When disaggregated by species, 77.5% of sheep (107/138; 95% CI 70.7–84.5) and 48.7% of goats (19/39; 95% CI 33–64.4) were seropositive (Table 1). The chi-square test yielded χ^2^(1) = 12.35, *p* < 0.01, indicated a statistically significant association between the presence of FMDV antibodies and species. Sheep were 3.63 times more likely to test positive for FMDV antibodies compared to goats (OR = 3.63, 95% CI 1.7–7.69). Sera with FMDV-specific antibodies were detected in all the LGAs investigated, as well as in all SR flocks that were intermixed with cattle where FMD outbreaks had been reported. In sheep, all villages investigated in this study showed presence of antibodies against FMD-NSP, ranging from 50 to 100%. A similar situation was noted for goats, except for Pasankani, Kogom-Tah and Fier villages (Table 2). In households where sheep and goats were housed together, the proportion of NSP-ELISA positive samples was higher in sheep than in goats across the LGAs, except in Kanam LGA and a village in Pankshin LGA (Table 2).

**Table 1:** **FMDV antibodies and genomic RNA detection in sheep and goats from Plateau state, Nigeria**

Species	No. of serum samples tested	No. positive (NSP-ELISA)	No. of oral swab samples tested	No. positive (rRT-PCR)
Sheep	138	107 (77.5%, 95% CI 70.7–84.5)	66	26 (39.4%, 95 CI 27.6–51.2)
Goats	39	19 (48.7%, 95% CI 33–64.4)	5	0 (0.0%)
Total	177	126 (71.2%, 95% CI 64–77)	71	26 (36.6%, 95% CI 25–49)

χ^2^(1) = 12.35, *p* < 0.01.

**Table 2:** **Proportion of small ruminants at household levels positive for FMD-NSP antibodies**

LGA	Village	Date of sample collection	Species	No. serum sample tested	NSP-ELISA positive	Proportion (%)
Qua’anpan	Doka	1/10/2020	Sheep	5	5	100.0
			Goat	5	4	80
Kanke	Tabulong	6/10/2020	Sheep	5	4	80
			Goat	5	4	80
	Myalche	9/12/2021	Sheep	6	3	50
Bokkos	Mabang	7/10/2020	Sheep	5	4	80
			Goat	5	3	60
	Magi-Mangor	8/10/2020	Sheep	5	4	80
	Faram	8/10/2020	Sheep	5	3	60
	Mangor-gida	8/10/2020	Sheep	5	3	60
	Pasankani	4/12/2020	Sheep	7	5	71.4
			Goat	6	1	16.6
Bassa	Bishe	13/10/2020	Sheep	9	8	88.9
	Mista Ali	16/10/2020	Sheep	10	9	90
Jos-South	Kogom-Tah	10/10/2020	Sheep	5	4	80
			Goat	5	1	20
	Zawan	30/9/2021	Sheep	12	10	83.3
		21/10/2021	Sheep	6	3	50
Wase	Bayan Dutse	14/10/2020	Sheep	10	5	50
	Angwan Kuyanbana	14/10/2020	Sheep	6	6	100
	Bayan Dutse	8/9/2021	Sheep	6	5	83.3
Shendam	Lougrel	15/10/2020	Sheep	10	10	100
Pankshin	Tambes Nomadic	4/11/2020	Sheep	10	8	80
			Goat	5	5	100
	Fier	12/11/2021	Sheep	6	5	83.3
			Goat	2	0	0
Kanam	Guiwa	27/11/2020	Sheep	6	4	66.7
			Goat	6	5	83.3

FMDV genomic RNA was detected in 36.6% (95% CI 25–49) of SR oral swab samples tested (Table 1). Viral RNA was identified in 26 of 66 (39.4%, 95% CI 27.6–51.2) oral swabs collected from sheep flocks mixing with cattle during FMD outbreaks in all nine LGAs. None of the five oral swab samples taken from goats tested positive for FMDV RNA. The rRT-PCR results from oral swabs showed Ct values ranging from 20.6 to 33.3, with 16 samples falling within the 20.6 to 29.4 range, reflecting the relative abundance of viral genomic material in the samples. Additionally, FMDV RNA was found in nine epithelial tissue samples obtained from six sheep and three goats displaying clinical lesions across four LGAs. Among these samples, five (three from sheep and two from goats) induced cytopathic effect (CPE) within 48 h in one or two passages in cell culture. Using antigen ELISA on the cell-cultured isolates, two FMDV serotypes were identified: O in sheep and A in goats, respectively. Within the households, the serotypes identified in SR epithelial tissue samples corresponded with those found in cattle samples using Ag-ELISA (Table 3).

**Table 3:** **Results of virus isolation and antigen-ELISA for FMDV from sheep and goats in reported outbreaks in cattle herd from Plateau state, Nigeria**

LGA	Date of sample collection	Species/No. of samples	Serotyping results by Ag-ELISA
Qua’anpan	01/10/2020	Cattle × 10 Goat × 3	A A × 2
Kanke	06/10/2020	Cattle × 8 Sheep × 1	O O
Bassa	13/10/2020	Cattle × 5 Sheep × 3	O O × 2
Pankshin	04/11/2020	Cattle × 2 Sheep × 2	A No CPE

**Figure 2:**
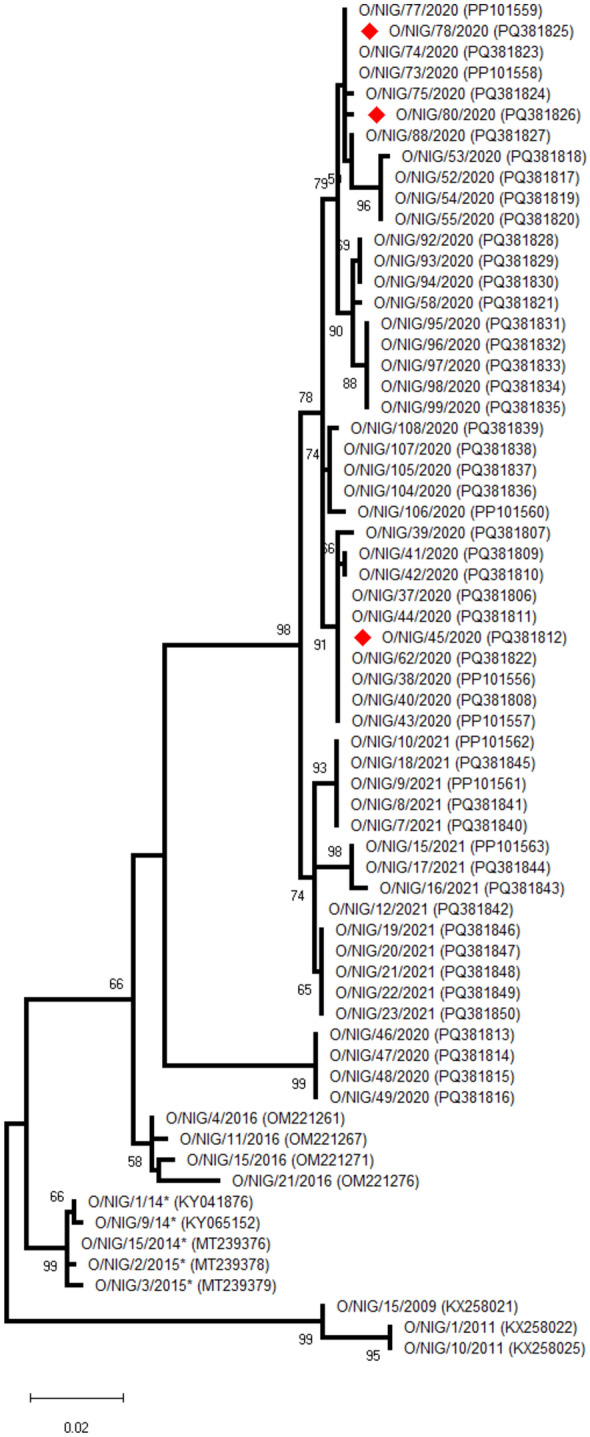
**Mid-point-rooted neighbor-joining tree showing relationship between FMDV serotype O isolates from sheep in Nigeria based on complete VP1 sequence.** Full length VP1 sequences of additional serotype O viruses from cattle in the same households with the sheep and those available in GenBank were included in the analysis (EA-3, East Africa-3).

**Figure 3:**
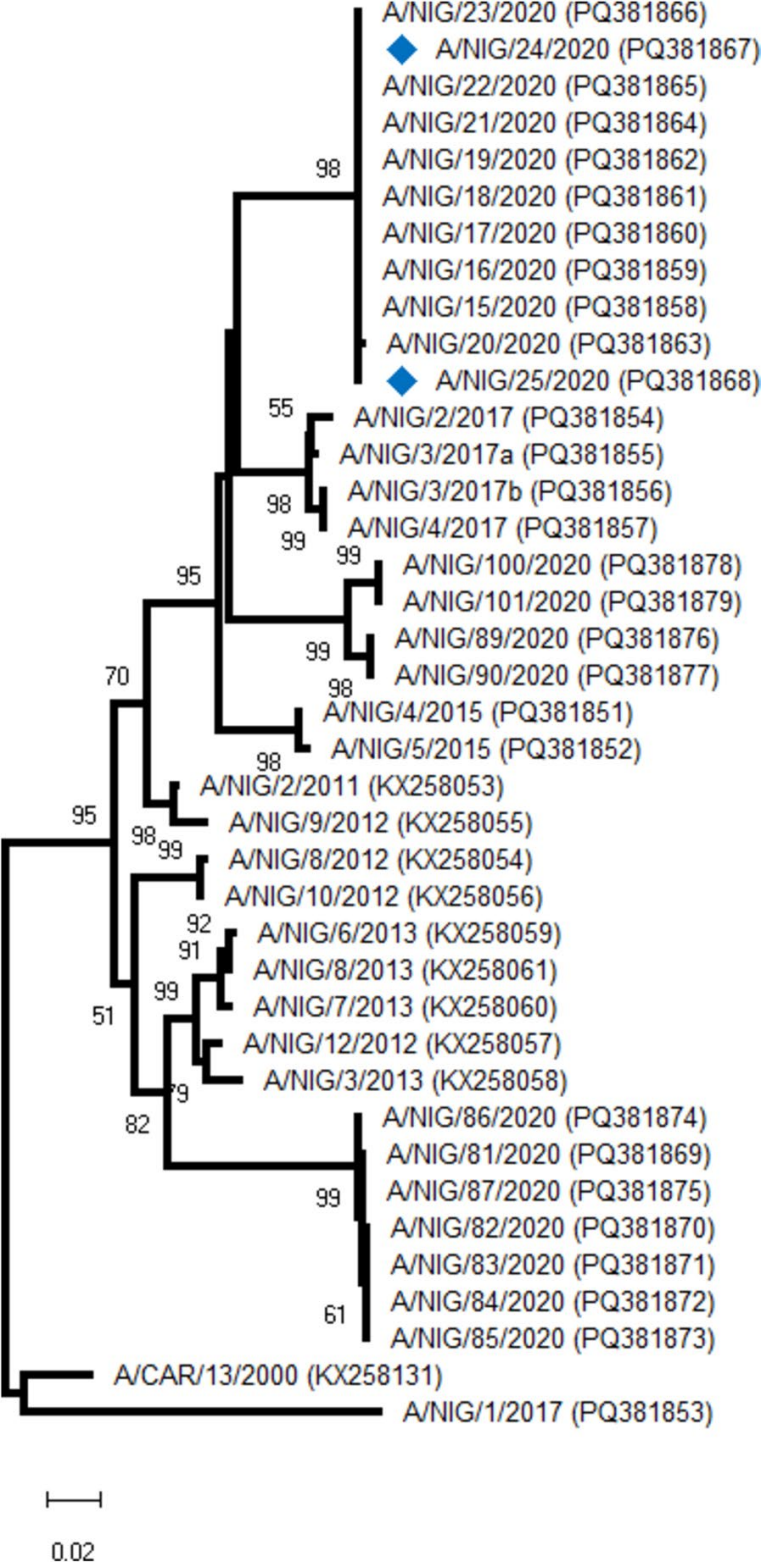
**Mid-point-rooted neighbor-joining tree showing relationship between FMDV serotype A isolates from goats in Nigeria based on complete VP1 sequence**. Full length VP1 sequences of additional serotype A viruses from cattle in the same households with the goats and those available in GenBank were included in the analysis.

The VP1 region of FMDV was amplified and sequenced from five isolates. The VP1 coding sequences of three FMDV serotype O viruses, obtained from sheep in Kanke and Bassa LGAs, were closely related and to FMDV isolated from cattle on the same farm during the same period. The phylogenetic analysis indicated that the FMDV serotype O viruses from sheep were most closely related to previous reported serotype O strains from Nigeria belonging to the EAST AFRICA 3 (EA-3) topotype (Figure 2). Two FMDV serotype A isolates found in goats during the outbreak in Qua’anpan LGA belonged to genotype G-IV within the AFRICA topotype. The sequences for the isolates showed high nucleotide identity (99.2–100%) to those obtained from cattle grazing communally in the same area. The phylogenetic analysis of the VP1 coding sequences of the serotype A viruses isolated from goats revealed that they clustered within subgroup G-IV, alongside with Nigerian isolates from 2012, 2013, 2015 and 2017 (Figure 3).

## Discussion

The aim of this study was to investigate the role of SR in the epidemiology of FMD in Nigeria. In most cases where sheep and goats tested positive in sera and oral swabs, symptoms or lesions typical of FMD were not detected. The main clinical signs noted in sheep and goats included fever, lameness and depression, while vesicles in and around the mouth, on the feet, or elsewhere were less frequently observed. The lack of clear clinical signs supports the findings reported by Kitching and Hughes [3] highlighting the difficulties to study the role of SRs in the epidemiology of FMD.

All sheep and goats that tested positive for FMDV-NSP were all in close contact with infected cattle, and every seropositive herd had at least one seropositive small ruminant. In a study conducted during outbreak investigations in cattle herds in four states in northern Nigeria, a similar percentage of sheep (64.4%) were seropositive [11]. In contrast, a lower proportion of seropositive SR (27.8%) was reported following FMD outbreak in Bauchi state, Nigeria [20]. Serosurveys conducted in SRs across Nigeria have reported a lower proportion of FMDV-NSP, ranging from 15 to 17.7% [17, 18, 31]. In serosurvey studies sampling a subset of the population, there is likely lower viral circulation within and between species than in outbreak situations, which could account for the discrepancies in sero-prevalence estimates compared to those recorded during an outbreak.

The percentage of FMD-NSP antibody positive sheep (77.5%) surpassed that of goats (48.7%) across the examined LGAs, except in Kanam and Pankshin LGAs. This pattern, in which sheep exhibit higher seropositivity compared to goats, aligns with findings from previous research conducted in Nigeria [17, 20] and India [32]. In Nigeria, the study revealed an FMD-NSP seroconversion of 41.66% for sheep and 21.81% for goats in one study [20] and 21.0% for sheep and 16.6% for goats in a later study with significant differences across states [17], while in India, the figures were 20.35% for sheep and 13.60% for goats. Conversely, a study in East Africa (Kenya) reported nearly identical levels of FMD-NSP antibodies in both sheep (24.0%) and goats (21.9%) [33]. Further studies to better understand potential reasons for these differences and implications for FMD epidemiology are needed.

In a parallel study, the force of infection estimated from age-specific seroprevalence data was lower in goats than in cattle and sheep, but similar between sheep and cattle [21]. This finding supports our observations and suggests that sheep, like cattle, may play a more important role in the transmission dynamics of FMD than goats, particularly within mixed-species production systems where close contact facilitates viral spread. A widespread tradition in livestock management involves the communal grazing of cattle, sheep, and goats, as well as cohabitating them on the same premises, observed in northern Nigeria, however in some areas is common practice to segregate goats and keeping sheep and cattle together.

The estimated proportion of detectable RNA by rRT-PCR in sheep from this study was lower compared to a similar study conducted in four states of northern Nigeria [11]. However, in the same report, a lower proportion of RNA was recorded compared to the present study, for sheep in close contact with cattle 1–3 months post-outbreak. In a cross-sectional study conducted in a grazing reserve in Kachia, Kaduna state, Nigeria, the detection of FMDV RNA (0.6%) by rRT-PCR in sheep sera was notably lower [18]. Furthermore, this study reports a much higher proportion of FMDV detectable RNA in sheep (36.6%) compared to the 2.0% and 2.6% reported in sheep and goats, respectively, from oral swab samples tested in households with reported FMD outbreaks in cattle [21]. Similarly, a lower proportion of viral RNA (2.2% and 1.1% for sheep and goats, respectively) was recorded for sera tested. The testing of oral swabs for FMDV RNA provides reliable evidence of an active infection within specific geographic area. This highlights the conclusion drawn by Gubbins and colleagues regarding the effectiveness of using oral swabs as viable sampling techniques for promptly detecting FMDV infection at both the individual animal and herd levels [33]. FMDV could not be successfully isolated in cell-culture from clinical material obtained from three sheep and one goat that were positive for FMDV RNA by rRT-PCR. This finding is consistent with previous reports indicating reduced success in virus isolation from samples with higher Ct values (> 25) [18], which may reflect lower quantities of detectable viral genetic material.

FMDV O/EA-3 and A/AFRICA/G-IV were identified in sheep and goats, respectively. Serological typing of serum samples has revealed that FMDV serotypes O and A, and to some extent SAT2 are the most prevalent serotypes in SRs in Nigeria [11, 18]. Upon comparing sequences obtained from SR samples with those from other cattle samples collected during outbreaks in same farms during the same period, we observed a close relationship between sequences obtained from SR samples and those from clinical samples in cattle. These findings do not clarify the direction of infection (whether it is the cattle infecting the small ruminants on the same farm or vice versa). However, movement of infected animals and animal products, and inadequate biosecurity measures further facilitate intra- and interspecies FMDV transmission. From an epidemiological viewpoint, research has demonstrated that sheep could play a significant role in the spread of FMD outbreaks, especially when infected sheep, showing no obvious clinical symptoms, are moved during the initial phases of an FMDV introduction into a previously unaffected area [34].

FMDV serotype O viruses from sheep were closely related to the serotype O strains identified from cattle in the same farms and to those previously reported in Nigeria between 2014 and 2016. Similar situation was also observed with the serotype A viruses reported in this study, however, they were also closely related to viruses previously reported between 2015 and 2017 in Nigeria. This finding provides further evidence of intra-state mobility of FMDV, potentially attributed to the pastoralist husbandry system and trade practices. Previous studies on isolation and identification of FMDV serotypes/strains in Nigeria and, other endemic countries in sub-Saharan Africa have primarily focused on outbreaks predominantly reported in cattle. To our knowledge, there has been no published report on the isolation and identification of FMDV in SRs in Nigeria and other endemic countries in West and Central Africa. This investigation provides the first documented record of FMDV isolation and identification in SRs in Nigeria and provides an insight on the role of these animals in the epidemiology of FMD in an endemic country.

## Conclusion

This study successfully isolated and characterized FMDV from small ruminants in Nigeria, marking an important advancement in our understanding of the epidemiology of FMD in this region. The findings also reveal that sheep have a higher seropositivity to FMDV than goats. These insights enhance our comprehension of FMDV dynamics in small ruminants and highlight the importance of integrating these results into comprehensive surveillance and control strategies. Ongoing research is crucial for monitoring FMDV prevalence and developing effective vaccination programs, ultimately supporting improved livestock health and agricultural productivity in Nigeria.

## Data Availability

The datasets analyzed during the current study are available from the corresponding author upon request.
